# Engagement With Conversational Agent–Enabled Interventions in Cardiometabolic Disease Management: Protocol for a Systematic Review

**DOI:** 10.2196/52973

**Published:** 2024-08-07

**Authors:** Nick Kashyap, Ann Tresa Sebastian, Chris Lynch, Paul Jansons, Ralph Maddison, Tilman Dingler, Brian Oldenburg

**Affiliations:** 1 Baker Department of Cardiovascular Research, Translation and Implementation La Trobe University Melbourne Australia; 2 Centre for Research Excellence in Digital Technology to Transform Chronic Disease Outcomes National Health and Medical Research Council Melbourne Australia; 3 Institute for Physical Activity and Nutrition School of Exercise and Nutrition Sciences Deakin University, Geelong Melbourne Australia; 4 Baker Heart and Diabetes Institute Melbourne Australia; 5 School of Psychology & Public Health La Trobe University Melbourne Australia; 6 Department of Medicine School of Clinical Sciences at Monash Health Monash University, Clayton Melbourne Australia; 7 Delft University of Technology Delft Netherlands

**Keywords:** cardiometabolic disease, cardiovascular disease, diabetes, chronic disease, chatbot, acceptability, technology acceptance model, design, natural language processing, adult, heart failure, digital health intervention, Australia, systematic review, meta-analysis, digital health, conversational agent–enabled, health informatics, management

## Abstract

**Background:**

Cardiometabolic diseases (CMDs) are a group of interrelated conditions, including heart failure and diabetes, that increase the risk of cardiovascular and metabolic complications. The rising number of Australians with CMDs has necessitated new strategies for those managing these conditions, such as digital health interventions. The effectiveness of digital health interventions in supporting people with CMDs is dependent on the extent to which users engage with the tools. Augmenting digital health interventions with conversational agents, technologies that interact with people using natural language, may enhance engagement because of their human-like attributes. To date, no systematic review has compiled evidence on how design features influence the engagement of conversational agent–enabled interventions supporting people with CMDs. This review seeks to address this gap, thereby guiding developers in creating more engaging and effective tools for CMD management.

**Objective:**

The aim of this systematic review is to synthesize evidence pertaining to conversational agent–enabled intervention design features and their impacts on the engagement of people managing CMD.

**Methods:**

The review is conducted in accordance with the *Cochrane Handbook for Systematic Reviews of Interventions* and reported in accordance with PRISMA (Preferred Reporting Items for Systematic Reviews and Meta-Analyses) guidelines. Searches will be conducted in the Ovid (Medline), Web of Science, and Scopus databases, which will be run again prior to manuscript submission. Inclusion criteria will consist of primary research studies reporting on conversational agent–enabled interventions, including measures of engagement, in adults with CMD. Data extraction will seek to capture the perspectives of people with CMD on the use of conversational agent–enabled interventions. Joanna Briggs Institute critical appraisal tools will be used to evaluate the overall quality of evidence collected.

**Results:**

This review was initiated in May 2023 and was registered with the International Prospective Register of Systematic Reviews (PROSPERO) in June 2023, prior to title and abstract screening. Full-text screening of articles was completed in July 2023 and data extraction began August 2023. Final searches were conducted in April 2024 prior to finalizing the review and the manuscript was submitted for peer review in July 2024.

**Conclusions:**

This review will synthesize diverse observations pertaining to conversational agent–enabled intervention design features and their impacts on engagement among people with CMDs. These observations can be used to guide the development of more engaging conversational agent–enabled interventions, thereby increasing the likelihood of regular intervention use and improved CMD health outcomes. Additionally, this review will identify gaps in the literature in terms of how engagement is reported, thereby highlighting areas for future exploration and supporting researchers in advancing the understanding of conversational agent–enabled interventions.

**Trial Registration:**

PROSPERO CRD42023431579; https://tinyurl.com/55cxkm26

**International Registered Report Identifier (IRRID):**

DERR1-10.2196/52973

## Introduction

Cardiometabolic diseases (CMDs) are a group of interrelated conditions, including heart failure and diabetes, that increase the risk of cardiovascular and metabolic complications [1]. The number of Australians managing CMDs is increasing, necessitating new approaches to better manage these conditions [2,3]. Approximately one-quarter of people globally are estimated to be living with metabolic syndrome and approximately 1 in 13 are living with cardiovascular disease [4,5]. The self-management of CMDs requires individuals to adhere to treatment regimens such as taking prescribed medications, maintaining a healthy diet, and performing regular physical activity to effectively manage their condition [6]. As these lifestyle modifications can be complex and ongoing, digital health interventions have emerged as an integral part of a scalable and accessible strategy to support individuals self-managing CMD [7,8].

In CMD contexts, the prolonged course of illness, often spanning decades, increases the difficulty of sustained engagement with self-management, where the risk of relapse remains a significant concern over time [9,10]. Therefore, it is essential that interventions aiming to improve self-management must be able to sustain user engagement over long time periods [10]. Indeed, low engagement rates undermine the effectiveness of digital health interventions; a recent review found an average participant dropout rate of 43% in studies assessing digital health interventions applied to the self-management of chronic diseases [11].

A recent systematic review identified improving personalization and interactivity as effective approaches to improving long-term engagement rates of digital health interventions [12,13]. This is particularly crucial in managing CMDs, where ongoing engagement and sustained behavior change are necessary for the effective attenuation of CMD complications [10]. As components within digital health interventions, conversational agents could greatly enhance the effectiveness of these self-management interventions by contextualizing information, offering constructive feedback, and fostering critical reflection [14-18]. A conversational agent is a technology that interacts with people using natural language, whether text-based or spoken, enabling accessibility to broader populations, including people with motor or cognitive disabilities [19]. Use of conversational agents could also reveal a linguistic dimension to a person’s health status, which could improve the personalization and effectiveness of digital health interventions [16,20].

Within the emerging research field of conversational agent–enabled interventions, the majority of existing tools are text-based, driven by machine learning algorithms, and delivered through mobile apps [21]. The literature on conversational agent–enabled interventions that support individuals with chronic diseases, including CMDs, to manage their condition is mainly composed of qualitative studies, quasiexperimental studies, pilot tests, and a limited number of randomized controlled trials, which all primarily examine prototypes of these interventions [22-25]. In clinical contexts, mental and physical wellness are the primary domains for the application of conversational agents, where they often provide emotional support [26,27]. In contrast, conversational agents applied to CMD self-management tend to provide a modality for monitoring symptoms and to deliver patient education [26]. However, recent trends indicate a shift from these basic functions to more complex and long-term end points in future applications, such as motivating behavior change [21]. This shift has occurred in parallel with technological advancements in cloud computing and transformer models, which have enabled a new generation of conversational agents termed large language models.

Large language models use deep neural networks to handle complex language tasks such as summarizing, generating, and translating natural language [28]. These tasks are achieved not by large language models understanding the prompts but rather by repeatedly predicting the word that is statistically the most likely to follow a sequence of words given as a prompt until a full response is offered [29,30]. However, this predictive approach means that large language models are potentially unsafe in clinical contexts, as they are liable to misinterpret prompts or generate unforeseen and inaccurate content [31-33]. Additionally, the opacity of this predictive approach hinders the ability to anticipate all possible outputs, making it challenging to establish safeguards that effectively prevent the dissemination of harmful or misleading information [34,35]. Furthermore, integrating these models into health care systems can involve transmitting sensitive, patient-identifiable data to third-party servers for processing, thereby imposing significant legal and ethical risks on health care providers [36,37].

Evidence indicates that the design features of conversational agent–enabled interventions impact the engagement of people with mental health issues in managing their condition [38,39]. For example, studies indicate that specific design features such as enhancing the anthropomorphic qualities of conversational agent–enabled interventions can improve engagement by reducing the monotony of repeated interactions [38,40]. Additionally, a recent review explored how design features pertaining to conversational architecture, such as delaying responses, proactive dialogues, and self-disclosures, affected user perceptions of conversational agents [41]. However, to date, there has not been a synthesis of evidence pertaining to conversational agent–enabled intervention design features (eg, personality) and their impacts on the engagement of people managing CMDs. In filling this gap, this review will contribute to the broader developer community making better informed choices when developing conversational agents, leading to more engaging, and therefore effective, conversational agent–enabled interventions for CMD self-management.

## Methods

### Protocol and Registration

This protocol is reported in accordance with the *Cochrane Handbook for Systematic Reviews of Interventions* and the PRISMA-P (Preferred Reporting Items for Systematic Reviews and Meta-Analysis Protocols) checklist [42,43]. The protocol for this review was registered in the International Prospective Register of Systematic Reviews (PROSPERO; CRD42023431579) in June 2023.

### Eligibility Criteria

#### Population

The review will include studies of adult participants (aged≥18 years) with a CMD diagnosis. CMDs are a group of interrelated conditions, including heart failure and diabetes, that increase the risk of cardiovascular and metabolic complications [1]. Populations reported with comorbidities such as mental health disorders or multiple CMDs will also be included.

#### Intervention

The review will include studies reporting on conversational agent–enabled interventions to assist people with CMD in managing their condition, such as by offering emotional or educational support and assisting individuals to monitor their symptoms.

#### Outcomes

The review will include studies reporting engagement outcomes such as ratings, interviews, analytics, and focus groups.

#### Study Design

The review will include primary research studies, including qualitative studies, quasiexperimental studies, observational studies, and randomized controlled trials. Reviews, editorials, protocols, and non-English publications will be excluded.

### Information Sources

We systematically searched the Ovid (Medline), Web of Science, and Scopus databases for relevant articles from inception until April 2024. Ovid (Medline) was selected owing to its medical and health science focus, which is useful for capturing research on the topic of CMDs. Web of Science and Scopus were both selected for their broader focus, which is useful for capturing research on conversational agents and engagement topics. To ensure a manageable scope, several databases were ruled out during preliminary searches. For example, Embase was ruled out because it had redundancy with Medline. In addition, the Cochrane Library was too specific, with 4 of the 5 exemplar papers present in all other databases not found in the Cochrane Library database. As this is a rapidly developing field, final searches will be conducted prior to the submission of a manuscript and additional studies that meet the inclusion and exclusion criteria will be incorporated into the review. Reference list searches will be conducted on all articles included in the full-text review.

### Search Strategy

An extensive set of search terms will be used related to the three central topics of the review: CMD, conversational agents, and engagement. Boolean operators will be used to combine search terms, including the following search string: (“Cardiovascular Diseases”[MeSH Terms] OR metabolic) AND (“conversational agent*” OR chatbot*) AND (accept* OR perceived). This approach is intended to yield a comprehensive collection of literature that explores the intersection of these central topics. An example of a complete search strategy is provided in Multimedia Appendix 1.

### Data Management

All search results will initially be imported into PaperPile, followed by removal of duplicate entries. The deduplicated library will then be exported from PaperPile and imported into Covidence (Veritas Health Innovation). Covidence (cloud-based software) will be used to store PDF files of articles considered during the full-text review. Additionally, Covidence will be used to store data extraction and quality appraisal forms, study selection results, and reviewer comments. Covidence will be used for data extraction and management and then the data will be exported to Microsoft Excel.

### Study Selection

During initial screening, two reviewers (NK and ATS) will independently examine the titles and abstracts of all studies collected from the search strategy. Assessments will be based on the defined inclusion and exclusion criteria. If conflicts arise, they will be settled during meetings between the two reviewers. If a conflict is not able to be resolved in this manner, the matter will be arbitrated by a third reviewer to achieve consensus.

During full-text screening, the remaining studies will be independently assessed by two reviewers to determine inclusion or exclusion using defined inclusion and exclusion criteria. Any conflict between the two reviewers will be settled during meetings, with arbitration by a third reviewer when required.

### Data Extraction

Data extraction from all included studies, including any supplementary material, will be performed and documented by two independent reviewers. Where more detailed information is required, authors will be contacted once for clarification.

The data extraction form will be designed to capture a wide array of details necessary to achieve the outcomes described above, primarily by capturing the perspectives of people with CMD on the use of conversational agent–enabled interventions. These details will include bibliographic information, study design, participants and population, intervention, and design features in terms of engagement outcomes.

### Quality Appraisal

The following Joanna Briggs Institute critical appraisal tools will be used: Checklist for Qualitative Research for qualitative research [44], Checklist for Quasi-Experimental Studies for quasiexperimental studies [45], and Assessment of Risk of Bias for Randomized Controlled Trials for randomized controlled trials [46]. The quality of evidence assessment results will be presented in a summary of findings table.

### Data Synthesis

An adapted version of the thematic synthesis analysis method developed by Thomas and Harden [47] will be used. This method will focus on data extracted from studies that detail design features and engagement outcomes. During the initial phase, these data will be categorized under multiple domains. Following the extraction, these domains will be iteratively consolidated until further merging would detract from the descriptive accuracy of the domains regarding their respective data sets. Subsequently, within each primary domain, the data will be further sorted into subdomains in a similar manner of iterative consolidation. Tables will be used to detail various aspects of the extracted studies, including study design, population, conversational agent characteristics, and design feature domains. Analysis of these themes will contribute to informing design choices for conversational agent–enabled interventions and to identify research gaps in how engagement tends to be reported in the literature.

## Results

**Figure 1:**
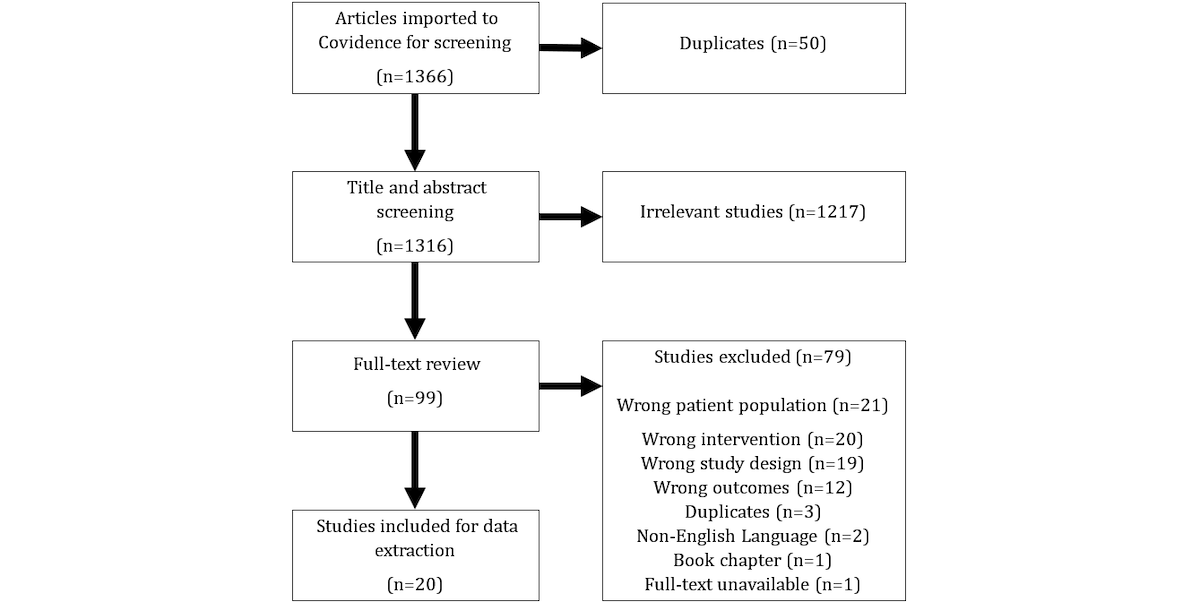
Flowchart illustrating the study selection process.

The review was initiated in May 2023 and, prior to title and abstract screening, was registered with PROSPERO in June 2023. Full-text screening of articles was completed in August 2023, followed by data extraction. Final searches were conducted in April 2024 (Figure 1) and the completed review was submitted to *Digital Health* in July 2024. Data pertaining to study design and sample size have been extracted and are summarized in Table 1.

**Table 1:** Characteristics of included studies.

Reference, publication year	Study design	Conversational agent role
Apergi et al [22], 2021	Quasiexperimental	To ask the patients the same series of questions related to their heart failure treatment and symptoms and provide feedback
Balsa et al [48], 2019	Qualitative	To assist older people with type 2 diabetes mellitus in medication adherence and lifestyle changes
Balsa et al [49], 2020	Qualitative	To support older people with type 2 diabetes mellitus in medication adherence and lifestyle changes
Baptista et al [23], 2020	Quasiexperimental	To deliver self-management education and support to adults with type 2 diabetes mellitus
Cheng et al [24], 2018	Qualitative	To provide a less cumbersome way for older patients with type 2 diabetes mellitus to effectively adhere to guidelines
Echeazarra et al [50], 2021	Randomized controlled trial	To help patients with hypertension self-monitor their blood pressure
Epalte et al [25], 2023	Qualitative	To counsel, educate, and train patients and family members with stroke with regard to rehabilitation, care, and other related issues
Gingele et al [51], 2023	Qualitative	To evaluate patients’ health status, provide patient education, and enable communication with heart failure nurses
Gong et al [52], 2020	Randomized controlled trial	To provide more accessible and engaging self-management support, monitoring, and coaching to adults with type 2 diabetes mellitus in Australia
Guhl et al [53], 2020	Quasiexperimental	To augment patient-centered health care by providing health education, monitoring, and problem-solving for users
Kimani et al [54], 2016	Quasiexperimental	To provide education on atrial fibrillation and promote adherence to daily heart rhythm monitor readings
Magnani et al [55], 2017	Quasiexperimental	To promote education, motivation, and monitor patient symptoms and adherence to behaviors
Roca et al [56], 2021	Quasiexperimental	To improve medication adherence in patients with comorbid type 2 diabetes mellitus and depressive disorder
Sagstad et al [57], 2022	Qualitative	To educate women with gestational diabetes mellitus
ter Stal et al [58], 2021	Qualitative	To support users in the self-management of chronic diseases in a long-term, daily life setting
Tongpeth et al [59], 2018	Qualitative	To improve patients’ knowledge of and response to acute coronary syndrome symptoms
Tsai et al [60], 2022	Qualitative	To support patients with chronic kidney disease manage their condition
Zhang et al [61], 2015	Qualitative	To counsel patients on their diagnoses and medications specified by a clinician, as well as increasing physical activity, improving diet, decreasing stress, and motivating them to be more involved and proactive in their own care

## Discussion

The effectiveness of digital health interventions in supporting people with CMD is dependent on the extent to which users engage with the digital tool [17,18]. Augmenting digital health interventions with conversational agents, technologies that interact with people using natural language, may enhance engagement because of their human-like attributes [24,25,57]. This protocol outlines a systematic review that aims to synthesize evidence pertaining to conversational agent–enabled intervention design features and their impacts on the engagement of people managing CMDs. The anticipated outcomes of the analysis include the identification of specific design features or themes within various domains that improve engagement with digital health interventions. Additionally, the quality appraisal process is expected to uncover research gaps on conversational agent–enabled interventions, thereby providing a clearer direction for future studies applied to investigating the design of conversational agent–enabled digital health interventions.

This synthesis will provide guidance on how best to embed engagement strategies within these interventions, thereby facilitating more engaging and effective strategies for supporting people managing CMDs. Additionally, characterizing the literature, in terms of how engagement tends to be reported, will have the benefit of identifying research gaps and highlighting areas for future exploration. These outcomes can in turn support researchers in developing a greater understanding of user engagement with conversational agent–enabled interventions.

## Appendix

Multimedia Appendix 1 Search strategy.

## Abbreviations

CMD: cardiometabolic disease
PRISMA: Preferred Reporting Items for Systematic reviews and Meta-Analyses
PRISMA-P: Preferred Reporting Items for Systematic reviews and Meta-Analyses Protocols
PROSPERO: International Prospective Register of Systematic Reviews
