# Supplementary material for: Engagement With Conversational Agent–Enabled Interventions in Cardiometabolic Disease Management: Protocol for a Systematic Review
Source: JMIR Res Protoc. 2024 Aug 7;13:e52973. doi: 10.2196/52973 (PMC11339562; doi:10.2196/52973)
Supplement: Multimedia Appendix 1 [file resprot_v13i1e52973_app1.docx]

Appendix 1

**Search strategy**

“Cardiovascular Diseases”[MeSH Terms] OR metabolic OR diabetes OR diabetic OR "metabolic syndrome" OR dyslipidemia OR dyslipidaemia OR "insulin resistance" OR "glucose intolerance" OR "impaired glucose" OR "high cholesterol" OR hyperlipidemia OR hyperlipidaemia OR hypercholesterolemia OR hypertriglyceridemia OR obesity

AND

"conversational agent*" OR chatbot* OR "virtual agent" OR "virtual assistant" OR "automated speech recognition" OR "relational agent" OR "embodied conversational agent" OR "virtual assistan*" OR "dialog* system" OR "digital assistan*" OR avatar*

AND

accept* OR perceived OR perception OR useful OR inten* OR use OR using OR attitude OR satisfy* OR producti* OR competen* OR adopt* OR resist* OR influence OR social OR rated OR rating OR adher*
